# Associations between prenatal alcohol and tobacco exposure on Doppler flow velocity waveforms in pregnancy: a South African study

**DOI:** 10.1186/s12884-023-05881-2

**Published:** 2023-08-23

**Authors:** Deborah Jonker, Brigitte Melly, Lucy T. Brink, Hein J. Odendaal, Dan J. Stein, Kirsten A. Donald

**Affiliations:** 1 Department of Paediatrics and Child Health, University of Cape Town, Cape Town, South Africa; 2 Department of Psychiatry and Mental Health, University of Cape Town, Cape Town, South Africa; 3 Department of Obstetrics and Gynaecology, Stellenbosch University, Parow, South Africa; 4 Neuroscience Institute, University of Cape Town, Cape Town, South Africa; 5 Unit on Risk and Resilience in Mental Disorders, South African Medical Research Council (SAMRC), Cape Town, South Africa

**Keywords:** Blood flow, Doppler ultrasound, Umbilical artery, Uterine artery, Middle cerebral artery, Prenatal alcohol exposure, Prenatal tobacco exposure, Birth weight

## Abstract

**Background:**

The negative impact of prenatal alcohol and tobacco exposure (PAE and PTE) on fetal development and birth outcomes are well described, yet pathophysiologic mechanisms are less clear. Our aim was to investigate (1) the associations between quantity, frequency and timing (QFT) of PAE and PTE with blood flow velocities in arteries of the fetal-placental-maternal circulation and (2) the extent to which combined effect of QFT of PAE and/or PTE and Doppler flow velocity waveforms (FWV) predict infant birth weight.

**Methods:**

The Safe Passage Study is a cohort based in urban Cape Town, South Africa. Recruitment occurred between 2007 and 2015. Information on QFT of PAE and PTE was collected prospectively at up to 4 occasions during pregnancy using a modified Timeline Follow-Back approach. Ultrasound examinations consisted of Doppler flow velocity waveforms of the uterine, umbilical (UA) and fetal middle cerebral arteries for the pulsatility index (PI) at 20–24 and 34–38 weeks. Exclusion criteria included: twin pregnancies, stillbirths, participants exposed to other drugs. The sample was divided into three groups (controls, PAE and PTE) and included 1396 maternal-fetal-dyads assessed during the second trimester; 1398 assessed during the third trimester.

**Results:**

PTE was associated with higher UA PI values in second and third trimesters (p < 0.001), compared to the PAE and control group. The total amount of cigarettes smoked during pregnancy was positively correlated with UA PI values (r = 0.087, p < 0.001). There was a positive correlation between cigarettes smoked per day in trimester one (r = 0.091, p < 0.01), and trimester two (r = 0.075, p < 0.01) and UA PI (in trimester two), as well as cigarettes smoked per day in trimester two (r = 0.058, p < 0.05) and trimester three (r = 0.069, p < 0.05) and the UA PI in trimester three. Generalized additive models indicated that PAE in trimester two, PTE in trimester one and Doppler FWV in trimester three were significant predictors of birth weight in this sample.

**Conclusion:**

In our study, PTE in trimesters two and three resulted in increased vascular resistance of the placenta. These findings highlight nuance in associations between PAE, PTE and blood flow velocities in arteries of the fetal-placental-maternal circulation and birth weight, suggesting that quantity and timing are important factors in these relationships.

**Supplementary Information:**

The online version contains supplementary material available at 10.1186/s12884-023-05881-2.

## Background

Harmful prenatal substance exposure remains a global public health problem. Maternal consumption may result in detrimental effects on fetal development and subsequent child health. Alcohol and tobacco use by pregnant women has been well-described as being associated with poor fetal growth and increased risk of birth complications such as preterm delivery, stillbirth and sudden infant death syndrome (SIDS) [1, 2]. Fetal Alcohol Spectrum Disorders (FASDs) refer to a spectrum of neuro-developmental conditions associated with prenatal alcohol exposure (PAE). In a recent systematic review, Lange and colleagues [3] reported that of 187 countries, South Africa has the highest prevalence of FASDs, estimated at 111.1 per 1,000 population (95% CI: 71.1 to 158.4 per 1,000 population) in certain regions. Previously reported findings from the Safe Passage study highlighted a prevalence of 46.2% of pregnant women consuming some alcohol in the cohort [4], while in a nearby cohort, hazardous alcohol use was reported in 13% of mothers in the Drakenstein Child Health Study [5]. In the same two South African birth cohorts, prenatal tobacco exposure (PTE) was also highly prevalent [4, 6], with dual exposure further increasing risk for adverse birth outcomes [2, 7].

Although the potential adverse outcomes are known, the pathophysiology of fetal impairment associated with PAE and PTE remains poorly understood. One potential mechanism for how these exposures may impact fetal growth and other outcomes, is restricted blood flow in the vascular territories of the placenta. During pregnancy, hemodynamic changes are significant in order to fulfil the needs of the developing fetus [8, 9]. Doppler ultrasound is a non-invasive method to assess placental blood flow [10] and can be applied to determine the blood flow in the uterine artery (UtA) of the mother and the umbilical (UA) and middle cerebral arteries (MCA) of the fetus. Resistance to blood flow is increased in the UtA and UA when poor placentation occurs, [11] leading to higher velocimetry indices measured by the pulsatility index (PI). Although these Doppler waveform analysis parameters have been linked with adverse obstetric and neonatal outcomes (i.e., pre-eclampsia and fetal growth restriction) [12, 13], the sum of reported studies on placental blood flow and vascular resistance secondary to PAE and/or PTE is limited and the results are not consistent.

Research on blood flow velocities in arteries of the fetal-placental-maternal associated with PAE has been mostly conducted in nonhuman primates and rat models, noting the differences between human and nonhuman primate pregnancy, including but not limited to length of gestation and placental architecture. A recent systematic review done by Pintican and colleagues [14] recognized only five studies using Doppler Ultrasonography to investigate the effects of PTE on placental vascularization. Limitations of current PTE studies include inter-individual heterogeneity, variation in levels of exposure, lack of multiple measurements over time and/or different metrics for measuring blood flow effects. There remains a lack of consistency regarding which placental beds, if any, are affected. Also, given the large individual variability in patterns of PAE and PTE, modest evidence exists for the impact of quantity, frequency and timing (QFT) of exposures on probable pathways for the adverse fetal outcomes associated with PAE and PTE. Disentangling the consequences of each substance as well as the timing of exposure is confounded by the varying data collection methods as well as frequent co-occurrence of smoking and drinking. Placental health represents fetoplacental metabolism, in turn affecting birth weight amongst other outcomes [15]. Birth weight is considered an expression of the intrauterine environment and an important metric of maternal and fetal health. It is frequently viewed as a global index of perinatal and neonatal outcomes [16]. Studies verified a link between PAE or PTE and reduced birth weight [17, 18], yet little is known regarding the underlying mechanisms constraining fetal growth.

To address gaps in the literature, we used a South African birth cohort sample to investigate (1) associations between QFT of PAE and PTE with blood flow velocities in arteries of the fetal-placental-maternal circulation and (2) the extent to which combined effect of QFT of smoking and/or drinking and Doppler FWV predict infant birth weight.

## Methods

The longitudinal cohort Safe Passage Study, conducted by the Prenatal Alcohol in SIDS and Stillbirth (PASS) Network, is a unique international, community-linked, study. It was the first multi-site study of SIDS and stillbirth to integrate prospectively collected pregnancy exposure information with a range of biological information to investigate the role of PAE in the risk for SIDS, stillbirth, fetal alcohol syndrome and FASDs.

As part of the Safe Passage Study of the PASS Network at Stellenbosch University, South Africa, 7060 pregnant women were recruited from the Bishop Lavis and Belhar residential areas for the period occupying August 2007 - January 2015. These sites were selected on account of a historical reputation for having a high prevalence of PAE and SIDS, and the need was recognized to include populations where the marked ethnic and socioeconomic disparities in SIDS remain understudied. Gestational age was determined by earliest ultrasound before the second antenatal visit. Of the participants who enrolled prior to 24 weeks gestation (representing 40% of participants), seven in ten were randomly selected and were invited to participate in the embedded study. The embedded study included the collection of additional information such as biometry and Doppler ultrasound velocimetry of uterine and fetal vessels. Detailed information on the participants and the study design have been described by Dukes et al. [19]

To investigate the aims stated above, data collected during the eight-year period was applied to analyse physiological profiles of participants who participated in the embedded study. Participants were excluded from the current analysis on the basis of (1) multiple births, (2) stillbirths, and (3) prenatal exposure to other recreational drugs (i.e. methamphetamine and marijuana). Pregnant women living with HIV, although not excluded from the birth cohort, were excluded from our analysis because of their higher risk of low birth weight infants [20].

A timeline follow-back measurement (TLFB) was used to elicit detailed self-reported exposure information. A blank calendar is used by participants to record incidents unique to their lives during a specified time period prior to the interview. This type of retrospective tool allows the user to contemplate QFT patterns for specific substances (e.g., alcohol or tobacco). In order to improve the method for verifying alcohol exposure information, a feasibility study (N = 518) was conducted on the target population prior to the birth cohort. According to findings from the feasibility study that have not been published, women in the target population frequently share beverages and frequently use different container sizes than those described in the traditional TLFB. In addition, a statistically significant difference was found between the number of drinks reported in a typical week, as a result of a single question, and the number of standard drinks as determined by the traditional TLFB with a seven-day reference period. Therefore, the standard TLFB and associated aids were modified for the Safe Passage Study [4]. In addition, Himes and colleagues [21] carried out a validation study that revealed strong concordance between maternal reports using this approach and meconium biomarkers. Considering the low literacy levels of the populations under study, interviewer administration was used. Before conducting the first interview, the interviewers were thoroughly trained and tested for proficiency. Biannual quality reviews were conducted throughout the data collection period.

At the recruitment interview, exposure information was collected for the time around conception (15 days before and after last menstrual period (LMP)) and for 30 days prior to the participant’s last reported drinking day. At subsequent interviews, if the participant reported consumption since her previous visit, the reference period consisted of the 30 days prior to the last drinking day. Peri-conception (two weeks prior and two weeks following the LMP) alcohol intake information was also collected. Total grams of alcohol consumed per drink were calculated and converted into standard drinks using the National Institute on Alcohol Abuse and Alcoholism (NIAAA) definition of one standard drink equals 14 g of pure alcohol. The *quantity* of PAE was measured using the total amount of standard drinks consumed during pregnancy and the average number of drinks consumed per week. In the current dataset, total alcohol consumption also included total standard drinks consumed around the LMP (± 15 days). The *frequency* of PAE was measured using the total number of days with binge consumption (more than four drinks per sitting) as well as the number of drinks per drinking day. *Timing* of PAE identified the total amount of drinks consumed per trimester.

PTE information was also obtained up to four times during pregnancy in which women reported their smoking habits for their last reported smoking day and 30 days prior. The *quantity* of PTE was measured using the average number of cigarettes smoked per day and the total amount of cigarettes smoked throughout pregnancy, from LMP to time of delivery. The *timing* of PTE was calculated using the total amount of cigarettes smoked per trimester. There was no measure of frequency of PTE in each trimester.

To incorporate all aspects of fetal circulation, we obtained Doppler measurements on different vascular beds [22] at two intervals during pregnancy, specifically at 20–24 weeks and 34–38 weeks. Placental vascular resistance was evaluated with recorded Doppler Flow Velocity Waveforms (FVW) from the UtA and UA arteries. We adjusted baseline, pulse repetition frequency and wall filter for all Doppler measurements so that aliasing was eliminated and maximal trace size was displayed on the single screen display, filling at least 50% of the image. UtA assessment included sampling both left and right UtA within 1 cm and anterior to the apparent crossover of the UtA with the external iliac artery. The early diastolic notching status of each vessel was recorded and the mean UtA PI (left and right) was calculated. For UA assessment, a free loop of umbilical cord was identified, halfway between the fetal and placental ends. In order to confirm fetal quiescence, a 5 mm sample volume was placed in the lumen of one UA, including a portion of the vein with a monophasic flow pattern. MCA was assessed in a transverse axial view of the fetal head that was slightly more caudal than the biparietal diameter section without placing undue pressure on the head. MCAs of the proximal hemisphere were identified using colour Doppler as major lateral branches of the circle of Willis, coursing anterolaterally in the vicinity of the lesser wing of the sphenoid bone, between the anterior and middle cerebral fossas, and towards the transducer. Within the proximal half of the MCA, a 5 mm gate was placed over the lumen of the MCA [23]. Two dedicated ultrasonologists performed all the ultrasound examinations in accordance with a standard operating procedure. Mean UtA PI for a particular gestational age was calculated using the reference interval table in Gómez et al. [24] For the UA, the mean PI according to gestation age was calculated using the formula developed by Drukker et al. [25]. A raised UtA and UA PI indicate increased fetoplacental and maternal–placental resistance respectively [26]. The MCA PI quantifies the fetal cerebral blood flow. A lower PI on the fetal MCA indicates that blood is being redistributed to the fetal brain [27].

### Statistical analysis

The participants were divided into three groups for preliminary analyses of the general characteristics of the sample population: the control group (did not smoke or drink), PAE group (mothers who drank during pregnancy), and PTE group (mothers who smoked during pregnancy). Exposure outliers (greater than or less than three standard deviations from the mean) were excluded from the analysis.

All analyses were carried out using R (v4.2.0) and RStudio [28, 29]. Statistical significance threshold was defined as p < 0.05. Data were checked for normality and log transformations were performed on the data to meet statistical assumption criteria. R scores and p-values for Spearman’s correlations were obtained to determine whether there were significant correlations between any two variables. One-way analysis of variances (ANOVA) and post-hoc Tukey Honestly Significant Difference (HSD) tests were used to ascertain statistical significance among control, PAE and PTE groups if distribution was normal. For variables that were not normally distributed, a Kruskal-Wallis test using a right tailed Chi square (χ^2^) distribution and post-hoc Dunn’s Tests were used to test for significance.

UtA and UA PI outliers were compared against reference values for a particular gestational age to determine whether there were any outlier values and whether these could be attributed to the PAE or PTE groups, as well as whether there is an association with an “outlier” doppler value and birthweight. “Outliers” were defined as PI values greater than or less than two standard deviations from the mean (PI > ± 2SD), and statistically significant differences were calculated using a t-test.

A generalized additive model (GAM) was performed to further understand whether a combined effect of QFT of smoking and/or drinking and Doppler FWV results on birth weight was detectable in this sample [30]. The use of a GAM (rather than a generalised linear model) allowed for the inclusion of non-linear functions for the covariates. The GAM formula is as follows:

$$g\left(\mu \right) = \alpha +{\sum }_{j = 1}^{p}fj\left(Xj\right) + \epsilon$$

where g($\mu$) is a link function, $\alpha$ is the intercept, and *f*_*j*_(.) are functions of the covariates/predictor variables [31]. A negative binomial distribution was applied, and a mixed model approach was used to estimate the smoothing parameter (restricted maximum likelihood). A smoothing function was also applied to all continuous variables to allow for the greatest flexibility in the model. A stepwise manual backward elimination process was performed and factors with a p < 0.2 were retained and the model was re-run until a final set of variables provided the best variance explained/ r^2^ value combination before adding additional information to the model [30]. In the first model, child and doppler data were included. In the second GAM, substance use (drinking and smoking) was added to the final GAM from the first stage. As it is well recognized in the literature that other risk factors, for instance the mother’s physical and mental health [32, 33] and socioeconomic status [34, 35] may influence birth weight, we decided to add other potential covariates into the model. In the third GAM, details of the mothers physical and mental health were added to the remaining variables that survived the stepwise manual backward elimination process from the first two GAMs. The process was repeated again for socio-economic factors. Only variables that were significant in each of the GAMs were reported in the results. The list of variables available/ included in each of the stages is described in supplementary Table S1.

## Results

**Fig. 1:**
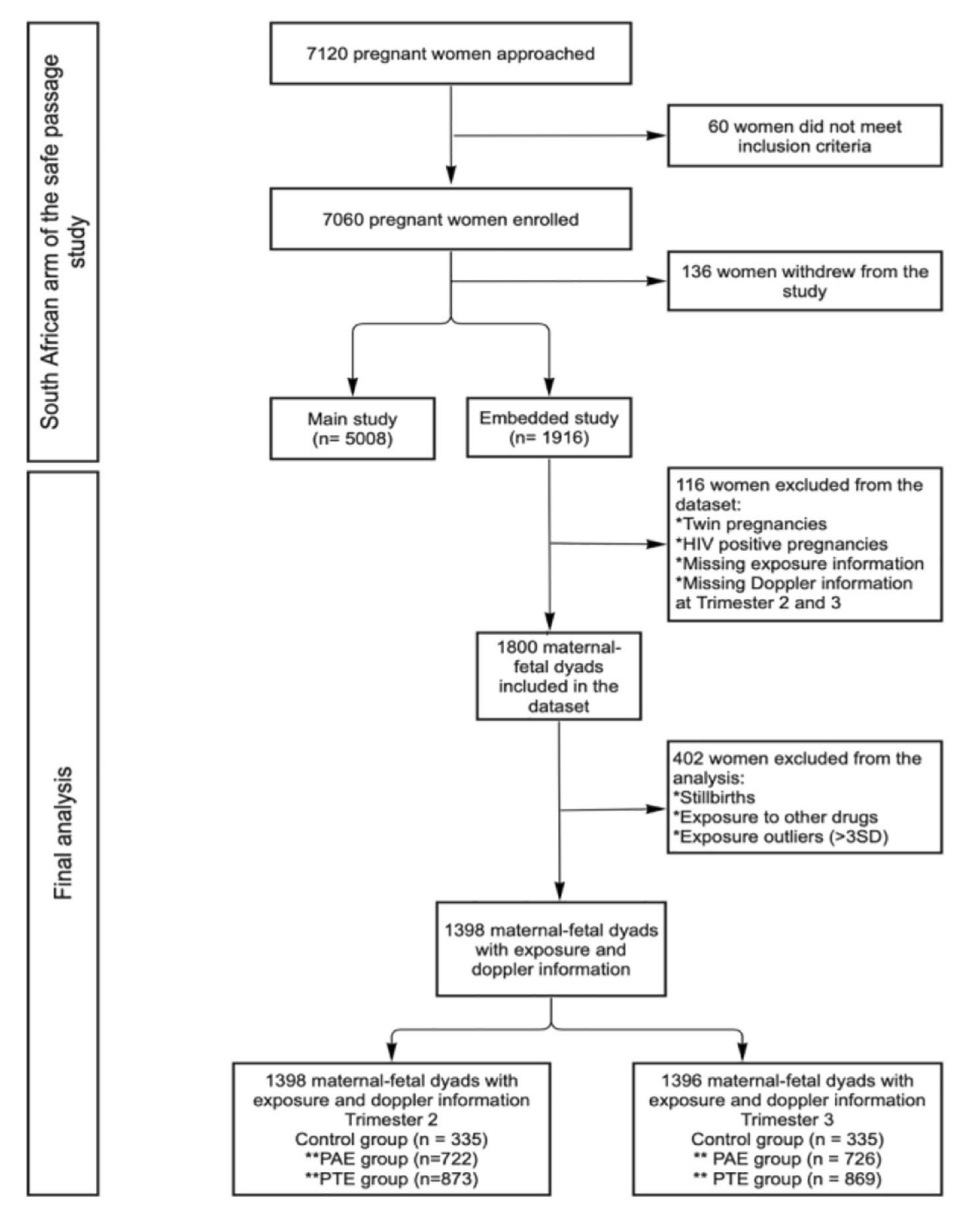
Flowchart outlining participants included in the final analysis List of acronyms: PAE = Prenatal alcohol exposure; PTE = Prenatal tobacco exposure. Note: * more than one reason may have been applied, **some samples fell into both PAE and PTE groups

The final sample consisted of a subset of 1396 maternal-fetal dyads assessed in the second trimester (22.64 ± 0.89 weeks gestation) and 1398 assessed in the third trimester (35.02 ± 1.01 weeks gestation. See Fig. 1 for a flowchart of participants included in the final analysis.

Distribution of participant characteristics (Table 1) is divided into three groups: controls, PAE and PTE. Across the three groups, the majority (74–76%) of mothers were between the ages of 20 and 35 years old, with an average age of 25 years (± 0.15 SE). Over 92% of the mothers had also finished primary school with most of them having some high schooling (72%), and this pattern was consistent across the three groups. Approximately half (51%) of the mothers had a normal (between 18.5 and 24.9) Body Mass Index (BMI). BMI scores were significantly higher in the control group (median = 25.53) compared to both drinking (median = 23.68) and smoking (median = 23.61) groups (Kruskal Wallis H χ^2^(2) = 23.04, p < 0.001; Dunn’s: p < 0.001). Across the three groups, over 88% of the mothers gave birth at full term ( > = 37 weeks gestation). There were differences between gestational age across the three groups (χ^2^(2) = 6.25, p = 0.044) and this was primarily between the control (median = 276 days) and PTE group (median = 274 days) (Dunn’s: p = 0.013). The PAE group had a median gestational age of 275 days. The proportion of male to female fetuses was well balanced across the three groups with 48–49% of the newborns being male. There was a significant difference in the birth weights across the three groups, with the highest average birth weight recorded in the control group (median = 3150 g), significantly higher than both the PAE (median 3040 g) and the PTE groups (median 3020 g) (χ^2^(2) = 21.13, p < 0.001Dunn’s: p < 0.001). There was a fairly low incidence of low birth weight in the whole sample, with only 14% of the newborns weighing less than 2500 g.

**Table 1:** Sociodemographic characteristics

Characteristics	PAE group (n = 848)	PTE group (n = 1011)	Control group (n = 335)
**Baseline characteristics**			
**Mean maternal age, years (± SD)**	24.6 ± 5.5	24.5 ± 5.7	25.6 ± 6.1
< 20	172	229	58
20–35	638	738	254
> 35	38	44	23
**Educational level**			
Any primary school	59	85	16
Some high school	565	698	193
Completed high school	187	183	100
Beyond high school	37	43	25
**Pregnancy Body Mass Index (BMI)**			
Underweight (BMI < 18.5)	53	84	14
Normal (BMI 18.5 - <25.0)	458	544	134
Overweight (BMI 25.0 to < 30.0)	176	200	77
Obese (BMI 30.0 to < 35.0)	78	95	53
Morbidly obese (BMI ≥ 35.0)	65	68	43
**Gestational age at delivery**			
< 28 weeks	13	17	3
28 weeks to 31 weeks 6 days	6	7	2
32 weeks to 36 weeks 6 days	84	106	29
> 37 weeks	745	881	301
**Infant characteristics**			
**Mean birth weight, grams (± SD)**	2951 ± 600	2938 ± 596	3129 ± 605
< 1500	21	24	4
1500 to < 2500	129	158	34
2500 to < 4000	672	799	272
≥ 4000	18	22	22
**Sex**			
Male	404	472	179
Female	444	539	156

Note: Data are presented as mean ± standard error. N represents total count

### Main effects of PAE and PTE on birth weight

The distribution of alcohol and tobacco exposure by trimester in terms of quantity and frequency is presented in Table 2. Within the PAE group, there was a large variation in the average number of standard drinks consumed during pregnancy of 20.8 ± 1.16 SE. Drinks consumed per drinking day, drinks consumed per week, binge count, and total number of standard drinks consumed during pregnancy all significantly decreased from the first to the third trimester (p < 0.001). In the PAE group, the mean birth weight of newborns was 2999 g ± 19 SE, and the gestational age of newborns was 38.9 ± 0.07 SE. There was also a significant negative correlation between the total number of drinks consumed during pregnancy and birth weight (r = -0.08, p < 0.001). The negative correlation between drinking and birth weight was strongest in trimester two with a negative correlation (r) of -0.13 (p < 0.001) for average drinks consumed per week. The same trend followed for drinks consumed per drinking day and total standard drinks consumed during pregnancy (r = -0.07; p < 0.05). There were no significant correlations between PAE and birth weight in the first and third trimesters in this cohort.

Within the PTE group, the average number of cigarettes smoked per day during pregnancy was 4.1 ± 0.11 SE. As with alcohol consumption, cigarette use also significantly decreased throughout the pregnancy (p < 0.001; Table 2). In the PTE group, the mean birth weight of newborns was 2970 g ± 18 SE, and the gestational age of newborns was 38.8 weeks ± 0.07 SE. There was a significant negative correlation between the number of cigarettes smoked per day and birth weight across all three trimesters during pregnancy (trimester one: r = -0.087, p < 0.001; trimester two: r = -0.097, p < 0.001; trimester three: r = -0.09, p < 0.001). There was also a negative correlation between the total number of cigarettes smoked during pregnancy and birth weight (r = -0.113, p < 0.001).

**Table 2:** Quantity and frequency of maternal alcohol and cigarettes used by trimester (timing)

Exposure variables	Trimester one (T1)	Trimester two (T2)	Trimester three (T3)	p-Value T1-T2	p-Value T1-T3	p-Value T2-T3
Drinkers (N)	683	550	268			
Non- drinkers (N)	875	997	1287			
Drinks consumed per drinking day	4.81 ± 0.16	3.29 ± 0.12	3.04 ± 0.16	p < 0.001	p < 0.001	p = 0.37
Drinks consumed per week	2.08 ± 0.19	0.93 ± 0.08	0.79 ± 0.07	p < 0.001	p < 0.001	p = 0.61
Binge count	1.41 ± 0.10	1.08 ± 0.11	0.64 ± 0.07	p < 0.001	p < 0.001	p = 0.07
Total number of standard drinks consumed	14.6 ± 1.06	10.6 ± 0.84	6.93 ± 0.52	p < 0.001	p < 0.001	p = 0.13
Smokers (N)	952	893	775			
Non-smokers (N)	611	653	778			
Number of cigarettes smoked per day	5.50 ± 0.15	4.19 ± 0.13	4.16 ± 0.13	p < 0.001	p < 0.001	p = 0.98

Note: Data are presented as mean ± standard error. N represents total count

Non-normal distribution of all three trimesters (using Shapiro Wilk Test), therefore significance tests were all done using Kruskal-Wallis H test. Post-hoc Dunn’s tests significance reported between two trimesters (T). Self-reported drinking and smoking were obtained at the recruitment interview, including up to three prenatal visits after recruitment, using a modified timeline follow-back interview for alcohol and tobacco use. Large standard deviations in the drinking variables are due to the number of drinkers consuming alcohol in some, but not all of the trimesters

### Associations of Doppler FWV with birth weight in substance use groups

There were negative correlations between both average UA PI (r = -0.184, p < 0.001) and average UtA PI (r = -0.315, p < 0.001) and birth weight in both the second and third trimesters (r = -0.220, p < 0.001; r = -0.206, p < 0.001, respectively). In addition, there was also a positive correlation between MCA PI in the third trimester and birth weight (r = 0.153, p < 0.001).

There were differences between UA PI in trimester two and both substance use groups (AOV (2,1807) = 5.15, p < 0.01). There were differences between the UA PI in the control group (mean = 1.19 ± 0.17) and the UA PI in the PAE (mean = 1.223 ± 0.17; post-hoc Tukey p < 0.05) and PTE groups (mean = 1.224 ± 0.17; post-hoc Tukey p < 0.01). Despite there being a negative correlation between PAE and birthweight in the second trimester (previous section), there were no significant differences between doppler data and birth weight in PAE and controls.

When looking at quantity-timing aspects of PTE, there was a positive correlation between the total number of cigarettes smoked during pregnancy and the UA PI in trimester two (r = 0.087, p < 0.001). There were also correlations between the number of cigarettes smoked in both the second and third trimesters and doppler data, with a positive correlation between cigarettes per day in trimester one (r = 0.091, p < 0.01), and trimester two (r = 0.075, p < 0.01) and UA PI (in trimester two), as well as cigarettes per day in trimester two (r = 0.058, p < 0.05) and trimester three (r = 0.069, p < 0.05) and the UA PI in trimester three. Infants born to mothers who both smoked and drank had a stronger correlation between UA PI and lower birth weights (r = -0.227, p < 0.001), compared to mothers that only smoked (r = − 0.189, p < 0.001) or drank (r = 0.181, p < 0.05).

Overall in the sample, abnormal UtA values (defined as PI ± 2SD) were seen in 7% fetuses in trimester two and 10% of fetuses in trimester three, and only 2% of these abnormal indices were seen in both trimester two and trimester three for the same fetuses. Abnormal UA values were observed in 19% of fetuses in trimester two and 10% in trimester three, and 3% had abnormal UA PIs in both trimesters. Abnormal Doppler (both UtA and UA) was seen in both the UtA and UA in 23 fetuses in trimester two and nine fetuses in trimester three (2% and 1% respectively). Birth weights were significantly lower in fetuses that had an abnormal UA PI in both second (t(87.68) = 6.50, p < 0.001) and third trimesters (t(146.3) = 5.66, p < 0.001). Birth weight and UtA PI showed similar differences, with significantly lower birth weights associated with abnormal UtA PI in the second trimester (t(330.3) = 2.76 p < 0.05). However, no significant differences were seen between abnormal doppler indices across the groups.

### Associations of PAE and PTE on Doppler FWV and birth weight

A stepwise manual backward elimination process was performed to further understand whether a combined effect of QFT of smoking and/or drinking and Doppler FWV results on birth weight was detectable in this sample. To mitigate the effects of gestational age on birth weight, weeks of gestation was included in the models. All the variables used in the models are listed in the tables below; however, only p values of significant factors (< 0.05) are reported in the table. The results of the final two GAMs are illustrated in Table 3; and all four GAMs are illustrated in the supplementary Table S1. Adding the mother’s other background variables only slightly improved the model (38.1 to 40.2%), with both a history of depression and hypertension in pregnancy negatively affecting birth weight. Adding socio-economic variables appeared to add complexity to the model without significantly improving model fit.

Doppler data collected in the third trimester appeared to be a more significant predictor of birth weight in the models, across all levels, especially MCA PI. Both UA PI and MCA PI came out as significant non-linear predictors in the models, which explains why they didn’t appear as significant in the correlations above. When exposure variables were introduced, only two variables came out as statistically important, smoking in trimester one and drinking in trimester two, which both significantly contributed to a lower birth weight.

**Table 3:** Independent variables included in the stepwise manual backward elimination (multi-level) GAM models for infant birth weight

	Covariates	GAM 3	GAM 4
**Child**	Gestational age at delivery in days	P < 0.001	P < 0.001
	Gender	P < 0.05	P < 0.01
**Doppler**	Uterine artery pulsatility index trimester 2		
	Umbilical artery pulsatility index trimester 2		
	Middle cerebral artery pulsatility index trimester 2		
	Uterine artery pulsatility index trimester 3		
	Umbilical artery pulsatility index trimester 3		P < 0.001
	Middle cerebral artery pulsatility index trimester 3	P < 0.01	P < 0.05
**Exposure**	Cigarettes per day trimester 1	P < 0.01	P < 0.05
	Cigarettes per day trimester 2		
	Cigarettes per day trimester 3		
	Total number of cigarettes smoked during pregnancy		
	Total number of standard drinks consumed trimester 1		
	Total number of standard drinks consumed trimester 2	P < 0.01	P < 0.05
	Total number of standard drinks consumed trimester 3		
	Total number of standard drinks consumed during pregnancy		
	Total number of binges during pregnancy		
	Variance explained/GAM results	40.2	40.7
	R^2^ adjusted	0.36	0.358

Note: A stepwise manual backward elimination process was run and the variables significant in the final model at each stage are indicated above

## Discussion

Several key findings emerged from this study: (1) There were no significant effects of community levels of PAE on blood flow velocities in arteries of the fetal-placental-maternal circulation in this cohort; (2) In contrast, PTE was associated with higher UA PI values in both the second and third trimesters compared to the PAE and control group, suggesting increased fetoplacental resistance. There were quantity-timing effects between the number of cigarettes smoked in both of these trimesters and UA PI values with a positive correlation demonstrated; (3) When taking multiple covariates into account, PAE in trimester two and PTE in trimester one appeared to more significant predictors of birth weight and (4) Doppler data collected during the third trimester surfaced to be a more significant predictor of birth weight in comparison to data collected during the second trimester. Third trimester data was demonstrated to be most significant in predicting birth weight in all samples, especially MCA PI.

In our sample, the group of infants with PAE had a significantly lower birth weight compared to the unexposed control group. Investigation of QFT within the PAE group revealed quantity-frequency effects on birth weight linked to specific timing. There were associations between the total number of drinks consumed during pregnancy (p < 0.05), average drinks consumed per week (p < 0.001), drinks consumed per drinking day (p < 0.05), with lower birth weight. However, these associations were mainly linked to maternal alcohol consumption during the second trimester, with first and third trimesters having a less clear association with lower birth weights in this cohort. Several studies have suggested that a dose-response relationship between PAE and birthweight exists, suggesting that heavy maternal alcohol consumption during pregnancy is associated with high risk of low birth weight [18, 36], while light or moderate alcohol consumption does not [37, 38]. Our findings suggest that this relationship is more nuanced, suggesting that both quantity and timing are important factors to consider in this relationship.

In this study PTE correlated with lower birth weight compared to control and PAE groups. There was a significant association between the number of cigarettes smoked per day and lower birth weight across all three trimesters during pregnancy (p < 0.001), but especially during the first trimester. This dose-dependent relationship between PTE and birth weight has been described in other studies in both middle- [6] and high-income countries [39]. Our results further support the importance of considering the quantity and timing of exposure in all substance-exposed pregnancies.

This study further reports an association between Doppler FWVs and birth weight. Our sample described negative correlations in the second and third trimesters between both the average UA PI and UtA PI values and birth weight (p < 0.001). There was a positive correlation between MCA PI in the third trimester and birth weight (p < 0.001). When we introduced other covariates such as hypertension and depression into the model, the fit improved, though slightly (38.1 to 40.2%), with both a history of depression prior to pregnancy and a history of pregnancy related hypertension in a previous pregnancy negatively affecting birth weight. Interestingly, by including socio-economic variables (i.e. employment, education, income, access to utilities), there was no significant improvement in the model. This is probably due to the lack of variability in these variables for the purpose of the broader study. Several maternal demographic and lifestyle characteristics have been correlated with adverse pregnancy outcomes. The effect of these characteristics on uteroplacental circulation might explain associations, but inadequate information is available about stated characteristics modulating placental resistance indices. The Generation R study (n = 7660), conducted in the Netherlands, described that women with higher maternal age, lower maternal educational levels and non-European descent tend to have slightly higher UtA and UA resistance levels from the second trimester onward, but these results were not consistent [40]. Folic acid supplements and parity were cited to influence placental resistance indexes. Another report (n = 7033) from the same cohort [41] indicated that educational level was strongly associated with the risk of continued high levels of UA PI from second to third trimester of pregnancy, with smoking being the factor that contributed the most to the association between education and placental resistance indices. When we examined the effect of possible demographic characteristics on Doppler indices (education, household income, access to utilities), none were significant in our sample. Future research is needed to investigate whether placental resistance indices are influenced by maternal demographic and lifestyle characteristics and whether they might be associated with increased risks of adverse pregnancy outcomes.

In our sample there were no demonstrated associations of PAE on arterial blood flow velocities in the fetal–placental–maternal circulation. Despite a strong negative correlation between the amount of alcohol consumed in the second trimester and a lower infant birth weight, there were also no differences in associations between Doppler data and birth weight between the infants of mothers in the drinking and non-drinking groups. Animal studies clearly demonstrate that alcohol freely crosses the placental barrier and enters the fetal circulation, with ovine [42] and baboon [43–47] models demonstrating that PAE acutely reduces fetal and maternal blood flow to the placenta in these animal models. Alcohol exposure has been shown in vitro to produce dose-dependent placental vasoconstriction and increase fetal-placental vascular resistance [48]. The lack of PAE effects in our sample may be contributed to our approach of including a population with a wide range of alcohol exposure. Thompson and Trudinger [49] applied a mathematical model of the umbilical placental circulation to study the effect of different physiological variables on the PI of the UA Doppler waveform. The outcomes indicated that significant increases in the UA PI only emerge after more than 60% of the placental terminal vascular branches are destroyed. Thus it is possible that using PI values is not an adequately sensitive measure of placental dysfunction when investigating community-levels of PAE. Binge and heavy chronic patterns of PAE may be more likely to result in demonstrable effects, a result often seen in other FASD-related pathophysiology studies [50].

In our cohort, PTE was associated with raised UA PI values in both the second and third trimester (p < 0.001), suggesting increased fetoplacental resistance. This is similar to other studies that have been conducted in middle (i.e. Turkey) and high-income countries (i.e. Netherlands, Poland), which also described an association between PTE and increased UA PI in both second [51] and third trimesters [52, 53]. Our present work validates previous findings showing significant quantity and timing effects of PTE on UA PI values. In our sample there were correlations between the number of cigarettes smoked and UA PI values in both the second and third trimesters. It has been mentioned that a significant increase in UA PI only occurs when ∼60% of terminal vascular branches are destroyed [49]. It is notable that even community-levels of PTE can have significant effects on fetoplacental vascular resistance, indirectly affecting birth weight. Little research has focused on mechanisms of tobacco consumption to be applied as a prognostic indicator for adverse neuro-behavioural outcomes, particular in the low and middle-income countries. Further research investigating the effect of QFT of PTE on fetoplacental vascular resistance and linking it to potential developmental impact would be highly relevant.

In the present study, the UtA PI values were consistent with the results of previous work (e.g., Yildiz et al. [54] (n = 79) and Alptekin et al. [53] (n = 148)) where no differences between chronic smokers (mothers who smoked five or more cigarettes per day) and non-smokers UtA PI values were found in the third trimester. Our finding of no significant changes in the MCA PI values secondary to PTE, is similar to that of previous work (e.g. Ates et al. [55] (n = 67), Yildiz et al. [54] and Alptekin et al. [53]) who demonstrated no differences between smokers’ and non-smokers’ MCA PI values in the third trimester. A study (n = 1120), embedded in the large prospective cohort Generation R study [52], on the contrary, observed that chronic PTE (mothers who smoked five or more cigarettes per day) was associated with an increased UtA PI as well as MCA PI in the third trimester. Some of these inconsistencies may reflect variance in the methodology used in these studies with a lack of consistency in the methods and parameters applied to measure uterine and placental perfusion. Other Doppler parameters such as the MCA/UA PI [56, 57] may also be more sensitive to assess the fetoplacental circulation subsequent to PTE than the MCA PI value alone.

One of the unexpected findings was the lack of association between the interaction of PAE and PTE on arterial resistance adaptations in this sample. This is in contrast to a previous study from the same cohort (n = 5806) that indicated that the PIs of the uterine and umbilical arteries were significantly increased in the high-drinking-high-smoking group in contrast to the single exposure groups [58]. In their study, high drinking constituted of four or more binge drinking episodes or 32 and more standard drinks and high smoking constituted of 10 or more cigarettes per day during pregnancy. This discrepancy is likely due to differences between study design and thus the potential relationship between the combined effect of PAE and PTE on maternal-fetal hemodynamic merits further investigation. Our findings suggest that the combined effects of PAE and PTE may be mechanistically different from mono-substance exposure, as it appears that there are non-linear responses occurring between the variables in our sample. When we investigated the combined effects of QFT of smoking and/or drinking and Doppler FWVs results on birth weight, two variables played a significant role: (1) number of cigarettes smoked per day in trimester one and (2) number of standard drinks consumed in trimester two, both associated with a lower birth weight. It has been proposed that PTE and PAE may interact synergistically to increase the risk of low birth weight [59]. Our model indicated that this relationship may be more complex than expected, highlighting the importance of understanding quantity and timing of exposure in studies of this type.

The major strength of our study is its prospective design from early fetal life and the size of the cohort. The Safe Passage Study draws participants from a homogenous ethnic and socioeconomic group and has employed precise measures to assess maternal drinking and smoking throughout all trimesters of pregnancy, allowing for novel analysis by leveraging QFT of exposure with prospective data. Although measurement error is inevitable based on self-reports, a study done by Himes and colleagues [21] indicated that the agreement between the Safe Passage Study Timeline Followback interview and neonate meconium alcohol marker ethyl glucuronide demonstrated 82% sensitivity (95% CI, 71.6-92.0%) and 75% specificity (95% CI, 63.2-86.8%) between PAE and ethyl glucuronide, suggesting that self-report methods using this approach are adequate.

Several limitations deserve emphasis. First, representativeness across cultures was limited for the South African cohort due to variations in drinking patterns, tobacco use, and environmental factors. Second, we included preterm births in this analysis, and thus prenatal exposure data might have been distorted by missed recruited visits due to prematurity. In a previous publication from the same cohort [60], women who delivered prematurely were found to consume more alcohol and smoke more tobacco. Infants born prematurely and hence missing their third trimester visit may also have an effect on the completeness of exposure data in this sample. Third, in our study, no information was collected concerning the time lapse between alcohol/tobacco use and ultrasonography; variation in this interval may have impacted our findings. Fourth, our analysis did not include doppler notching data, which is known to be an important marker for detecting increased vascular resistance during pregnancy. Based on a meta-analysis conducted by Sciscione and Hayes [61], a diastolic notch with a pathologic PI was the best predictor of an adverse pregnancy outcome. And lastly, the lack of significant associations of community levels of PAE, PTE, and their interaction with abnormal Doppler indices could be due to insufficient sample size or more complex/non-linear interactions that our methods did not assess.

## Conclusion

We identified that PTE in both the second and third trimesters is associated with increased vascular resistance of the placenta. Although community levels of PAE were not associated with increased vascular resistance, the potential relationship between the combined effect of PAE and PTE on maternal-fetal hemodynamic merits further investigation. Furthermore, when considering predictors of birth weight, PAE in trimester two, PTE in trimester one and Doppler data collected in trimester three appear to be most significant in this sample. The use of prospective QFT data extends our understanding of how important it may be to consider community levels of exposure when examining underlying pathophysiologic mechanisms contributing to adverse birth outcomes secondary to PAE and/or PTE.

### Electronic supplementary material

Below is the link to the electronic supplementary material.

Supplementary Material 1

## Data Availability

The datasets analysed during the current study are available from the corresponding author upon reasonable request and with the permission of Prof HJ Odendaal.

## Abbreviations

SIDS: Sudden infant death syndrome
FASDs: Fetal alcohol spectrum disorders
PAE: Prenatal alcohol exposure
PTE: Prenatal tobacco exposure
UtA: Uterine artery
UA: Umbilical artery
MCA: Middle cerebral artery
PI: Pulsatility index
QFT: Quantity, frequency, and timing
PASS: Prenatal Alcohol in SIDS and Stillbirth Network
TLFB: Timeline Follow-Back
NIAAA: The national institute on alcohol abuse and alcoholism, FVW:Flow velocity waveforms
GAM: Generalized additive model
BMI: Body mass index
