# Supplementary material for: Associations between prenatal alcohol and tobacco exposure on Doppler flow velocity waveforms in pregnancy: a South African study
Source: BMC Pregnancy Childbirth. 2023 Aug 23;23:601. doi: 10.1186/s12884-023-05881-2 (PMC10464169; doi:10.1186/s12884-023-05881-2)
Supplement: Supplementary file 1 — Supplementary Material 1 [file 12884_2023_5881_MOESM1_ESM.docx]

**Supplementary material**

**Maternal demographic and lifestyle characteristics**

Self-reported maternal characteristics (e.g., demographics and medical history) and information regarding maternal mental health during pregnancy was obtained at 20–24 weeks’ gestation. Depressive symptoms were measured using the Edinburgh Postnatal Depression Scale (EPDS), a depression screening tool developed to specifically assess depressive symptoms in perinatal women where higher scores indicate more severe depression symptoms. We used a cut-off of ≥13 to indicate maternal depression [1,2].

Depression was recorded in 45 mothers prior to pregnancy, with 84% of these occurring in the PAE and PTE groups (7 mothers in the control group). Accordingly, the average Edinburgh depression scale score was significantly lower in the control group (mean = 11.7+_5.9), falling below the referable threshold of above 13 (AOV (2,2153) = 5.8, p <0.005). Both the PAE and PTE groups recorded average Edinburgh depression scale scores of 12.9, with ca. 20% falling within the first threshold for referral to a social worker. In addition, approximately 25% of the mothers in both these groups had Edinburgh depression scores of above 18, the second threshold.

Hypertension data was not recorded for all the participants, but the number missing was proportional to the size of the group. Thus, trends are still apparent. Mothers in the control group were reported as having slightly higher pregnancy related hypertension scores with 15%, compared to ca. 10% in the PAE and PTE groups. This pattern was also observed in hypertension recorded in previous pregnancies with 10% in the control group and 5% in the substance use groups. However, hypertension (not pregnancy related) was highest in the PTE group (3.8%), followed by 3.2% and 2.9% in the PTE and control groups respectively.

**Table S1 Independent variables included in the stepwise manual backward elimination GAM models for infant birth weight**

|  | **Covariates** | **GAM 1** | **GAM 2** | **GAM 3** | **GAM 4** |
| --- | --- | --- | --- | --- | --- |
| **Child** | Gestational age at delivery in days | P < 0.001 | P < 0.001 | P < 0.001 | P < 0.001 |
|  | Gender | P < 0.001 | P < 0.001 | P <0.05 | P < 0.01 |
| **Doppler** | Uterine artery pulsatility index trimester 2 |  |  |  |  |
|  | Umbilical artery pulsatility index trimester 2 |  | P < 0.1 |  |  |
|  | Middle cerebral artery pulsatility index trimester 2 |  |  |  |  |
|  | Uterine artery pulsatility index trimester 3 | P < 0.1 | P < 0.1 |  |  |
|  | Umbilical artery pulsatility index trimester 3 | P < 0.001 | P < 0.001 |  | P < 0.001 |
|  | Middle cerebral artery pulsatility index trimester 3 | P < 0.001 | P < 0.001 | P < 0.01 | P < 0.05 |
| **Exposure** | Cigarettes per day in trimester 1 |  | P < 0.1 | P <0.01 | P < 0.05 |
|  | Cigarettes per day in trimester 2 |  |  |  |  |
|  | Cigarettes per day in trimester 3 |  |  |  |  |
|  | Total number of cigarettes smoked during pregnancy |  |  |  |  |
|  | Total number of standard drinks consumed trimester 1 |  |  |  |  |
|  | Total number of standard drinks consumed trimester 2 |  | P < 0.01 | P < 0.01 | P < 0.05 |
|  | Total number of standard drinks consumed trimester 3 |  |  |  |  |
|  | Total number of standard drinks consumed during pregnancy |  |  |  |  |
|  | Total number of binges during pregnancy |  |  |  |  |
| **Mother** | Edinburgh Depression scale |  |  |  |  |
|  | Depression prior to pregnancy |  |  |  | P < 0.1 |
|  | Pregnancy related hypertension |  |  |  |  |
|  | History of hypertension not pregnancy related, prior to pregnancy |  |  |  |  |
|  | History of pregnancy related hypertension in a previous pregnancy |  |  | P < 0.1 | P < 0.1 |
| **Household** | Education |  |  |  |  |
|  | Employment status |  |  |  | P < 0.05 |
|  | Household income (ZAR) |  |  |  |  |
|  | Type of house |  |  |  |  |
|  | Access to flushing toilet at home |  |  |  |  |
|  | Access to running water at home |  |  |  |  |
|  | Access to electricity at home |  |  |  |  |
|  | Variance explained/Gam results | 36.4 | 38.1 | 40.2 | 40.7 |
|  | R^2^ adjusted | 0.338 | 0.352 | 0.36 | 0.358 |

ZAR= South African Rand. Note: A stepwise manual backward elimination process was run and the variables significant in the final model at each stage are indicated above.

References

1. Dukes KA, Burd L, Elliott AJ, Fifer WP, Folkerth RD, Hankins GDV, et al. The safe passage study: design, methods, recruitment, and follow-up approach. Paediatr Perinat Epidemiol. 2014;28(5):455-65.
2. Shuffrey LC, Sania A, Brito NH, Potter M, Springer P, Lucchini M, et al. Association of maternal depression and anxiety with toddler social-emotional and cognitive development in South Africa: a prospective cohort study. BMJ Open. 2022:13;12(4):e058135. doi: 10.1136/bmjopen-2021-058135.
